# Extended-Spectrum-Beta-Lactamase (ESBL)-Producing *Escherichia coli* in Laying Hens: Slaughterhouse Prevalence and Antibiotic Resistance Patterns

**DOI:** 10.3390/antibiotics14040351

**Published:** 2025-03-31

**Authors:** Nihat Telli, Arife Ezgi Telli, Yusuf Biçer, Gamze Turkal

**Affiliations:** 1 Department of Food Processing, Vocational School of Technical Sciences, Konya Technical University, 42250 Konya, Turkey; ntelli@ktun.edu.tr; 2 Department of Food Hygiene and Technology, Faculty of Veterinary Medicine, Selcuk University, 42130 Konya, Turkey; yusufbicer@selcuk.edu.tr (Y.B.);

**Keywords:** antibiotic resistance, ESBL, *Escherichia coli*, laying hen, poultry slaughterhouses

## Abstract

**Background:** Laying hens, which are widely utilized for consumption and export in various regions, experience prolonged antibiotic exposure due to their longer lifespan, increasing the risk of antibiotic resistance and impacting the microbial environment of poultry slaughterhouses. Given the significance of extended-spectrum-β-lactamase (ESBL)-producing *Escherichia coli* in food safety, this study aimed to investigate the prevalence of ESBL genes in *E. coli* isolated from a laying hen slaughterhouse in Konya, Turkey. **Methods:** Sampling was conducted using a convenient sampling approach, and a total of 150 samples were collected from a single slaughterhouse over six visits during both warm (June–August) and cold (January–March) seasons to evaluate seasonal variations. Samples were categorized into environmental sources (personnel, air, wastewater, eggs) and carcass-related sources (cloaca, carcasses at critical control points, final product). Classical cultural and molecular techniques and antimicrobial susceptibility tests were used for ESBL presence and gene characterization. For sequence analysis, the bidirectional Sanger Gene sequence analysis method was applied. **Results:** PCR-based detection identified 10 of the 17 isolates as *E. coli* by amplifying the *usp*A gene, and bidirectional Sanger sequencing further confirmed these isolates at the species level. The *E. coli* isolates were detected at various sampling areas, including personnel, carcasses after evisceration, and raw wastewater samples collected at different time points. In the multiplex PCR analysis, most ESBL isolates were positive for the bla*CTX-M* gene. The co-existence of bla*TEM* and bla*CTX-M* genes was detected in five samples. Additionally, three genes (bla*SHV*, bla*CTX-M*, and bla*OXA*) were identified in a carcass sample after evisceration. All ESBL-producing isolates harbored the bla*CTX-M1* gene, and multiple antibiotic resistance was observed across all isolates. The presence of these genes was strongly associated with resistance to ampicillin, amoxicillin-clavulanic acid, aztreonam, cefepime, cefpodoxime, cefuroxime, and cephalothin, highlighting the critical role of bla*CTX-M* in driving the multidrug resistance patterns observed in this study. The highest resistance rate (80%) was observed in “personnel” and “carcass samples after evisceration”, while all isolates remained sensitive to carbapenems (imipenem and meropenem). **Conclusions:** Our findings highlight the importance of the laying hen slaughter line as a potential source of contamination with *ESBL*-producing *E. coli*, which poses significant implications for food safety and public health. These findings underscore the need for improved control measures to mitigate ESBL *E. coli* transmission in poultry processing and highlight the importance of optimizing antibiotic use strategies in laying hen farming.

## 1. Introduction

Poultry production, particularly laying hens, provides an important source of protein and plays a key role in global food security. However, the widespread use of antimicrobials for treatment and growth promotion in food animals, including cattle, pigs and poultry, raises concerns about antimicrobial resistance. The long-term exposure of laying hens to antibiotics for egg production increases the risk of developing resistance. These hens, which are also offered for meat consumption after the end of the laying cycle, may pose a greater risk of spreading resistant bacteria through the food chain.

The spread of antimicrobial-resistant *Escherichia coli* in farm animals, including poultry, pigs, and cattle, poses a significant public health risk due to potential transmission through the food chain [1]. Recent studies on this subject have reported the presence of resistance genes, including ESBL and colistin resistance, in *E. coli* isolated from various farm animal environments and have emphasized the role of excessive antimicrobial use in the development of resistance [1,2,3]. Given the complex interactions between humans, animals, and the environment, a One Health approach is essential to effectively address antimicrobial resistance and limit its spread across different ecosystems. In particular, genomic analyses of pathogenic *E. coli* isolated from poultry have shown various resistance mechanisms contributing to the persistence and spread of the microorganism, emphasizing the need for continuous surveillance in poultry production systems [2,3].

The unconscious use of antibiotics in healthcare treatment procedures has led to the spread of antibiotic-resistant bacteria, not only in living organisms, but also in the environment [1]. Currently, clinical cases reported with the development of resistance to β-lactam group antibiotics represent an important public health problem [4,5]. Although new β-lactam agents have been developed as a result of developments in pharmacology, it is accepted that the product diversity has become almost ineffective against the development of resistance. The most important mechanism of bacterial resistance to β-lactam group antibiotics is the production reaction of one or more enzymes that cause hydrolysis of the openable β-lactam ring in the chemical structure of the antibiotics. In terms of their properties, these enzymes are defined as β-lactamases [5]. Extended-spectrum β-lactamases are characterized as highly potent bacterial enzymes that exhibit resistance to β-lactam group antibiotics and often other antibiotic classes [4,6]. β-lactamase activity, which can be transferred between bacteria, is controlled by a large number of genes that have been classified [5,7]. The presence of ESBLs in various members of the *Enterobacteriaceae* family, especially *E. coli*, is of great microbiological and clinical importance [8]. Although initially ESBL production seemed to be limited to nosocomial infections caused by *Klebsiella pneumonia*, it is now mostly associated with community infections of the urinary tract caused by *E. coli* [4]. The failure of treatments and the potential threats posed by this situation require understanding the epidemiology of ESBL-producing bacterial infections and controlling their spread. In this spread, human carriers are the main reservoir for the community, through direct contact, but it may alternatively be associated with livestock [4].

The determination of resistance profiles of pathogens related to food of animal origin and their environment provides important contributions to the scientific explanation of the dynamic phenomenon of antibiotic resistance. Today, food animal production systems require the use of large amounts of antimicrobials for a variety of purposes, including disease control, prophylaxis, growth promotion, and especially the treatment of infections. The high prevalence of multi-resistant strains and antibiotic resistance genes in a wide range of environmental sources from which samples were obtained indicates an increase in the spread of commensal flora and foodborne pathogens [9]. Hu et al. (2016) suggested that the spread of antibiotic resistance among livestock and humans can be mostly attributed to the transfer abilities of mobile genes detected in *Proteobacteria*, *Bacteroidetes*, *Actinobacteria*, and *Firmicutes* species [10]. When the common mobile genes detected in isolates obtained from animals and humans were evaluated, it was determined that poultry was a more frequent source compared to other farmed animals [11]. Although there is less scientific data on laying hens, it has been revealed that an increase in the effects of antimicrobial resistance (AMR) in bacteria has been observed in humans after consumption of meat and eggs [12,13]. It is known that *E. coli* strains can be transmitted to humans both directly and through the food chain, and possible antimicrobial resistance in these strains can be transferred [12,13]. Moreover, wild birds, particularly migratory and captive species, have also been identified as important reservoirs of antimicrobial resistant E. coli*,* potentially contributing to resistance gene dissemination to poultry or livestock via direct contact or environmental contamination [14].

The aim of this study was to comprehensively address the flow and seasonal characteristics of AMR gene determinants in the laying hen slaughter and production line using ESBL-producing *E. coli* as a model. Considering that antimicrobial use along this breeding and production line is generally low compared to the broiler sector, other additional environmental factors need to be included in the scientific interpretation if resistance genes are to be detected.

## 2. Results and Discussion

### 2.1. Detection and Confirmation of the ESBL E. coli Isolates

The sampling points considered positive for ESBL isolates as a result of classical cultural and phenotypical ESBL antibiotic tests are given in Table 1. The sample points where ESBL-positive samples were detected, in order from most to least, are as follows: four from personnel, four from eggs, three from raw wastewater, four from carcasses after evisceration, and one each from carcasses after bloodletting and the final product samples. Overall, positive samples accounted for 11.33% (17/150).

**Figure 1:**
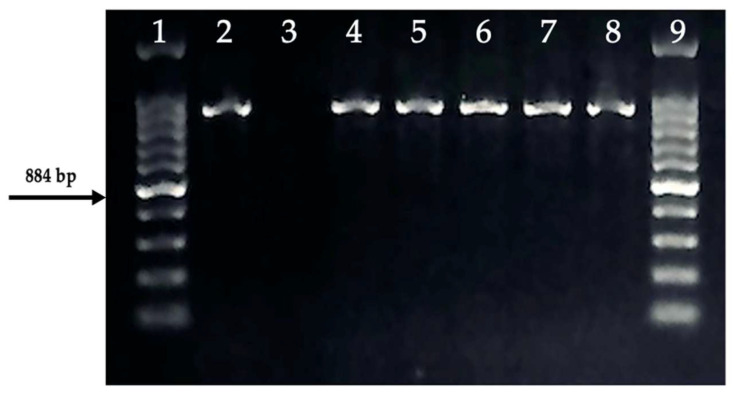
Positive samples with the *E. coli* species specific *usp*A gene region. 1, 9: 100 bp Ladder, 2: Positive Control ATCC 25922, 3: Negative Control, 4–8: Positive Samples.

Seventeen samples that were identified as positive through classical cultural analyses were subjected to PCR using *E. coli*-specific *usp*A gene primers. The PCR analysis revealed an 884 bp band corresponding to the *usp*A gene region in 10 of the 17 samples (Figure 1). Further confirmation of *E. coli* species identification was achieved through bi-directional Sanger sequencing of the *usp*A gene, with species identification confirmed based on sequence similarity to known *E. coli* strains in the National Center for Biotechnology Information (NCBI) database.

The Fisher’s Exact Test revealed no statistically significant association between seasons and positivity rates (*p* = 0.327). Despite this, a descriptive analysis of the seasonal distribution showed that the proportion of positive samples was higher during the warm period (9.3%; 7/75) compared to the cold period (4%; 3/75). It is known that environmental temperature is very important among the external factors affecting the development of microorganisms. However, numerous scientific data expressing the spread of microorganisms around the world show that the prevalence in relevant geographies is affected by factors such as seasonal temperature averages. Although there is a great diversity within the *E. coli* species due to its ability to survive in different environments, the optimum growth temperature for the microorganism is generally accepted as 37 °C. For Konya province (37°52′ N, 32°29′ E), the mean temperatures of June, July, and August between 1929 and 2022 were reported as 20.1, 23.5, and 23.3 °C (mean, 22.3 °C), respectively. For the same period, January, February and March had reported temperatures of −0.2, 1.5, and 5.5 °C (avg., 2.26 °C), respectively [15]. In the light of the findings and scientific literature, the high monthly temperature averages cause a parallel increase in the detection rate in terms of microorganism prevalence. Based on the months in which samples were collected, the positivity rates, listed from highest to lowest, were as follows: August (12%; 3/25), July (8%; 2/25), June and March (8%; 2/25), and February (4%; 1/25). Notably, no positive samples were detected in the January samples. This variation is likely attributable to the alignment between the sampling months and temperature averages that are closer to the optimal growth conditions for the microorganism. These findings are consistent with research by Khalid et al., who examined *E. coli* contamination in cloacal samples from various bird species across different seasons (spring, summer, autumn, and winter) [16]. Their findings suggested that *E. coli* contamination in game poultry is generally higher during the rainy summer months compared to the winter.

### 2.2. Detection of the ESBL Genes

**Figure 2:**
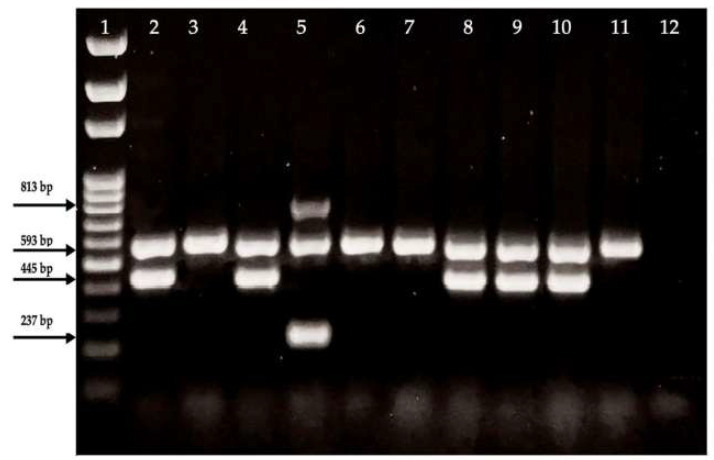
Multiplex PCR analysis of the ESBL genes: bla*SHV* (237 bp), bla*TEM* (445 bp), bla*CTX-M* (593 bp), and bla*OXA* (813 bp). 1: Ladder (100 bp); 2, 8, 9, 10: bla*CTX-M* and bla*TEM*; 3, 6, 7, 11: bla*CTX-M*; 4: bla*TEM*, bla*CTX-M*; 5: bla*CTX-M*, bla*SHV*, bla*TEM*; and 12: negative control.

The presence of ESBL-associated genes was confirmed by multiplex PCR. The UV transilluminator gel image of the multiplex PCR reaction used to detect the bla*SHV*, bla*TEM*, bla*CTX-M* and bla*OXA* genes in ESBL-producing *E. coli* is presented in Figure 2.

Multiplex PCR analysis revealed the presence of the bla*CTX-M* gene in all samples. Both the bla*TEM* and bla*CTX-M* gene regions were detected in five samples, while the bla*CTX-M* gene region was identified in only four samples. Additionally, three gene regions bla*SHV*, bla*CTX-M*, and bla*OXA*, were detected in a carcass sample after evisceration.

**Figure 3:**
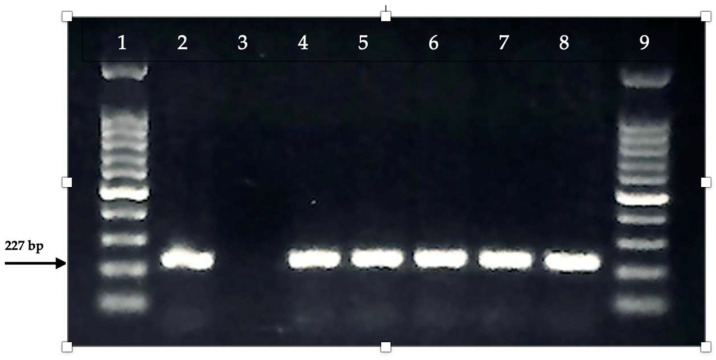
Multiplex PCR of the bla*CTX-M1*, bla*CTX-M2*, bla*CTX-M9*, bla*CTX-M8/25* genes. 1, 9: Ladder (100 bp); 2: Positive Control (*Klebsiella penumoniae* NCTC 13440); 3: Negative Control; 4–8: bla*CTX-M1* positive samples.

All isolates were found to carry the bla*CTX-M1* gene, while the bla*CTX-M2*, bla*CTX*-*M9*, and bla*CTX-M8/25* gene regions were not detected in any of them, as determined by multiplex PCR analysis of bla*CTX-M* group genes (Figure 3).

Similar studies in the poultry industry have reported the detection of various *CTX* genes in ESBL isolates. For instance, Wibisono et al. [17] reported that among 130 cloacal samples collected from four laying hen farms in Indonesia, 8.69% harbored ESBL-producing *E. coli*, and bla*CTX-M* genes were detected in 80% of the ESBL-positive isolates. In commercial laying hen litter materials from Spain, bla*CTX-M1* gene regions were detected in 19 of 141 isolates, while bla*CTX-M14* and bla*SHV-12* were found in 1 and 9 isolates, respectively. Additionally, bla*TEM-1B* was identified in two isolates, and bla*TEM-1D* in one isolate, as reported by Aldea et al. [13].

Additionally, a study from Malaysia reported an overall ESBL-producing *E. coli* prevalence of 37.2%**,** with blaCTX-M genes being the most dominant, particularly in poultry farm environments and chicken samples [18]. In Brazil, 66% of *E. coli* isolates from poultry farms were positive for ESBL production, with blaCTX-M group 2 being the most prevalent [19]. Similarly, a pan-European surveillance study found that 8% of *E. coli* isolates from chickens carried ESBL genes, with blaSHV-12 (32.3%), blaCTX-M1 (24.2%), and blaCMY-2 (22.2%) as the most common variants. These studies highlight the widespread presence of ESBL genes in poultry production systems across different regions [20].

In our study, bla*TEM* genes were detected in five isolates, and the bla*SHV* gene was identified in one isolate. In contrast, Effendi et al. [21] reported the absence of bla*SHV* genes in *E. coli* isolates from broiler cloacal samples in Indonesia. Additionally, their study detected bla*TEM* genes in 7 of the 10 isolates, showing a slightly higher prevalence compared to our findings. Consistent with these findings, Lemlem et al. [6] reported that 84.5% of *E. coli* isolates from Malaysian broiler chickens carried at least one ESBL gene, with bla*CTX-M* detected in 62.9% and bla*TEM* in 45.4%. Similarly, Faridah et al. [22] observed high β-lactam resistance (86.1%) among *E. coli* isolates from broiler cloacal swabs in Indonesia, though only 29.6% were phenotypically confirmed as ESBL producers. Among these, bla*CTX-M* was present in 32 of the 34 ESBL-positive isolates, bla*TEM* in 13, and 12 harbored both genes. The distribution of the ESBL genes is shown in Table 2.

Differences in ESBL types are generally related to the transferable plasmids of various species of Gr (-) bacteria and the ability of ESBL genes to be transferred horizontally to other bacterial species [23]. ESBL genes are located on plasmids that can be easily transferred between bacterial species. Some ESBL genes are mutant derivatives of resident plasmid-mediated β-lactamases (e.g., bla*TEM*/*SHV*), while others can be mobilized from environmental bacteria (e.g., bla*CTX-M*) [24]. However, it is reported in the scientific literature that antimicrobial resistant bacteria and their determinants can be transmitted from food animals to humans through direct contact and/or animal products [25]. Although fecal transport is widely recognized as the primary source of ESBL gene dissemination in the environment, foods can also serve as a potential reservoir given that bacterial species like *E. coli*, which are part of the normal gastrointestinal flora, may harbor these resistance genes [21,22]. The origin of *CTX-M* enzymes differs from *TEM* and *SHV* ESBLs. While *SHV* and *TEM* ESBLs are produced by amino acid substitutions of their parent enzymes, *CTX-M* ESBLs are obtained through horizontal gene transfer from other bacteria using genetic apparatus such as conjugative plasmids or transposons [26]. It has been reported that the prevalence of the *CTX-M* type extended-spectrum β-lactamase has recently increased significantly among *E. coli* clinical isolates isolated in Asia, Africa, Europe, and America. The rapid spread of *CTX-M* enzymes worldwide is facilitated by plasmids, transposons, and integron gene regions. In addition to plasmid-mediated transfer, *E. coli* can also acquire ESBL genes through additional horizontal gene transfer mechanisms, including transformation (uptake of free DNA from the environment) and transduction (bacteriophage-mediated transfer of resistance genes). While conjugation is the predominant route of ESBL gene dissemination, recent studies suggest that integrons, splice sequences, and bacteriophages may also contribute to the mobility and persistence of resistance determinants [27]. These mobile genetic elements integrate β-lactamase genes into stable genetic platforms, facilitating rapid adaptation of *E. coli* to antimicrobial pressure and increasing their transmission potential. Moreover, the stability of carrying ESBL-encoding plasmids affects bacterial survival and persistence, and some plasmids harbor compensatory mutations [28]. In poultry environments, continuous exposure to antimicrobials through therapeutic use or feed supplementation creates a selective pressure that favors the persistence and dominance of ESBL-producing *E. coli* [29].

Given the widespread presence of ESBL-producing *E. coli* in poultry farms and the potential risk of transmission, implementing effective control strategies is essential. To mitigate the prevalence of ESBL-producing *E. coli*, several intervention strategies have been proposed to mitigate the prevalence of ESBL-producing *E. coli* in poultry farms. Strengthening biosecurity measures within farms, such as improved sanitation, controlled farm access, and enhanced waste management, can reduce bacterial transmission [19]. Additionally, competitive exclusion strategies, such as the use of probiotics and bacteriophages, may help limit colonization by resistant strains, while responsible antimicrobial management remains essential for minimizing selection pressure [30].

### 2.3. Antibiotic Resistance Profile of the Isolates

Antibiotic susceptibility testing was conducted on all ESBL-positive samples, rather than only ESBL-producing *E. coli*, to capture additional resistance reservoirs and provide a more comprehensive understanding of β-lactam resistance dissemination. Antibiotic resistance test profiles of the ESBL isolates are presented in Table 3.

All isolates exhibited multidrug resistance to the 10 tested antibiotics belonging to the penicillins, carbapenems, cephalosporins, and fluoroquinolones groups. The most common profile of multiple resistance was amoxicillin–clavulanic acid, ampicillin, aztreonam, cefepime, cefpodoxime, cefuroxime, and cephalothin. The isolates with the highest resistance rate (80%) were found to be personnel^1^ and carcass^1^ samples after evisceration. All isolates exhibited resistance profiles to the antibiotics amoxicillin–clavulanic acid, ampicillin, aztreonam, cefepime, cefpodoxime, cefuroxime and cephalothin. In other words, resistance was detected against selected antibiotics from the aminopenicillin, monobactam, fourth generation cephalosporin, second generation cephalosporin, and first generation cephalosporin groups. However, all isolates exhibited resistance against cefpodoxime, a third generation cephalosporin. All isolates were found to be sensitive of the carbapenem group antibiotic, imipenem and meropenem. For the third generation cephalosporin group antibiotic moxalactam, resistance was detected in personnel^1^ and carcass^1^ after evisceration isolates. For moxalactam, moderate resistance was observed in 6 of 17 isolates (35%), including personnel^2^, personnel^3^, carcass^3^ (after evisceration), as well as egg^1^, egg^3^, and egg^4^ isolates. Research on similar sample groups has demonstrated that different antibiotics display varying resistance and susceptibility patterns. For example, Olopade et al. [31] reported that the highest resistance rates were observed against ampicillin, tetracycline, sulfamethoxazole–trimethoprim, gentamicin, and imipenem antibiotics. However, multiple resistance was observed in 78% of the isolates. In the light of the findings, the researchers stated that antimicrobial agents were used for therapeutic or prophylactic purposes in all sampled farms and that appropriate biosecurity measures were not taken. However, it has been emphasized that eggshells are potential reservoirs for multi-antibiotic resistant *E. coli* and ESBL-producing *E. coli*. The high resistance to ampicillin observed in this study is consistent with the findings of Olopade et al. [31]. However, there are differences regarding imipenem resistance. In a comparable study, Pais et al. [32] reported 57% of the isolates had multiple antibiotic resistance and 3.8% were positive for ESBL-producing *E. coli.* In our study, ampicillin exhibited the highest resistance rate. Additionally, nalidixic acid, tetracycline, ciprofloxacin, sulfonamide, trimethoprim, chloramphenicol, azithromycin, and ceftazidime were among the most resistant antibiotics. The isolates were 100% susceptible to cefotaxime and meropenem. The most common profile of multiple resistance (42.2%) was penicillin, fluoroquinolone, tetracycline, sulphonamide, and various agents. The ampicillin resistance findings in this study are consistent with those reported by Pais et al. [32]. Similarly, Wibisono et al. [33] aimed to determine the genotype profiles of ESBL-producing *E. coli* strains isolated from laying hen cloacal samples and the sensitivity patterns of phenotypic antibiotics on extended-spectrum β-lactamase genes. All ESBL-producing isolates were found to be resistant to amoxicillin, ampicillin, cefazolin, cefotaxime, and ceftriaxone. However, over 70% resistance was detected against gentamicin, aztreonam, and trimethoprim/sulfamethoxazole. In light of the findings, researchers highlighted that laying hens serve as a potential reservoir for ESBL-producing *E. coli*, underscoring their role in the dissemination of antimicrobial resistance. The resistance to amoxicillin, ampicillin, and aztreonam reported by Wibisono et al. [33] aligns with the findings of this study. In a more recent study, Bennameur et al. [34] reported that all 92 *E. coli* isolates obtained from farms exhibited multidrug resistance. The highest resistance rates were observed against tetracycline, ampicillin, and nalidixic acid, while 22 isolates were resistant to chloramphenicol, 4 to gentamicin, and all isolates remained susceptible to colistin. Currently, in terms of nonspecific types of antibiotic resistance, third and fourth generation cephalosporin resistance caused by extended-spectrum-beta-lactamase (ESBL)-production is considered an important public health problem [4]. However, it has been reported that the main source of the resistance observed against third and fourth generation cephalosporins in *E. coli* is due to broad-spectrum beta-lactamases, which are increasingly reported in isolates obtained from humans and poultry [13]. The detection of resistance to cefpodoxime and moxalactam (third generation) and cefepime (fourth generation) antibiotics in this study is considered significant in this context.

While this study provides valuable insights, it is important to acknowledge certain limitations. Unlike broiler production, research on laying hens remains relatively limited, particularly in terms of antimicrobial resistance surveillance. This is partly due to the smaller number of authorized slaughterhouses processing spent laying hens and the lower commercial focus on their meat. As a result, sample collection opportunities are more restricted, which may impact the generalizability of findings. Expanding research in this area is crucial to fully understand the antimicrobial resistance dynamics in laying hen production.

## 3. Material and Methods

### 3.1. Sampling Sites and Sample Collection

The laying hens sampled in this study were obtained from conventional farms, where birds were raised under standard conventional production systems. The hens included in the study had reached the end of their laying cycle and were sent for slaughter. In providing the samples, it was aimed to comprehensively represent the line from the shipment and transportation of the laying hens to be slaughtered from the farms and their acceptance into the business to the final product. For this purpose, samples were collected from pre-slaughter live animals, slaughterhouse critical control points, personnel, plant air, wastewater, and final products. A total of 150 samples were collected across six visits (25 samples per visit) during both hot and cold seasons, covering live animals, critical control points, personnel, air, wastewater, eggs, and final products. A power analysis was conducted for a two-group comparison (cold vs. warm seasons) to detect a medium effect size (Cohen’s d = 0.5) with 80% power (α = 0.05). The analysis suggests that approximately 64 samples per group (128 total) would be necessary for adequate statistical power.

Twenty-five samples [cloaca (n = 5), carcasses at critical control points of the slaughter line (after bloodletting (n = 2), after evisceration (n = 4), slaughter line personnel (n = 5, collected from individuals directly involved in processing activities within the slaughterhouse), air sampling (slaughter line air (n = 1), packaging–labeling department air (n = 1), cold storage room air (n = 1)), 1000 mL of raw wastewater (n = 1), 1000 mL of treated wastewater (n = 1), egg (n = 2), and final product (n = 2)] were collected in accordance with sampling regulations (ISO 18593:2018) [35]. Amies Swab Viscosa (Deltalab, Barcelona, Spain) was used to collect swab samples. Air samples were obtained using an active mechanism air sampler (Air Ideal, BioMerieux, Marcy l’Etoile, France) that measures 190 L of air with an absorption speed of ˂20 m/s and then blows it into the petri dishes [36]. The air sampler was placed on a suitable surface to sample for 1 h. Wastewater samples were taken into sterile bottles, at a rate of 250 mL every 60 min [37]. Egg and final product samples were transferred to sterile stomacher bags. All samples were transported to the laboratory under cold storage conditions and analyzed immediately.

### 3.2. Bacterial Isolation, Identification and Characterization

The samples were incubated in Buffered Peptone Water (BPW, Oxoid CM0509, Oxoid, Basingstoke, UK) and wastewater samples were incubated directly at 37 °C for 24 h under aerobic conditions. Afterwards, 100 µL was taken and inoculated on Brilliance ESBL Agar. After 24–48 h of incubation, colonies growing in chromogenic medium were distinguished according to their colors [38]. Plate Count Agar (PCA) medium (Merck 1.05463, Merck, Darmstadt, Germany) was used to determine the culturable aerobic mesophilic bacterial diversity in the air samples and they were then transferred to Brilliance ESBL Agar (Oxoid PO5302, Oxoid, Basingstoke, UK).

For confirmation of ESBL production, antibiotic disks of cefotaxime 5 μg or ceftazidime 10 μg (Oxoid, Basingstoke, UK) and cefotaxime/clavulanic acid and ceftazidime/clavulanic acid (bioMérieux, Marcy l’Etoile, France) E tests were used. A Cefpodoxime Combination Disc Kit (Oxoid, Basingstoke, UK) was used according to the manufacturer’s instructions. From each sample, 5 colonies considered suspicious were selected. Colonies grown on PCA were stored in Brain Heart Infusion Broth (BHI, Merck 110493, Merck, Darmstadt, Germany) supplemented with 15% (*v*/*v*) glycerol (Merck 104057, Merck, Darmstadt, Germany) at −20 °C for further analysis.

### 3.3. Molecular and Genotypical Characterization of Isolates

DNA extracted with a DNeasy Blood and Tissue Extraction Kit (Qiagen, Germantown, MD, USA) was used in PCR for identification and genotypic characterization of suspected isolates. The concentration and quality of the extracted DNA were determined with a nanodrop spectrophotometer (Titertek Berthold, Colibri, Germany). Genomic DNA was stored at −20 °C for further analyses.

#### 3.3.1. Molecular Identification of the *E. coli*

The presence of the species-specific *usp*A gene for *E. coli* (*usp*A F 5′CCGATACGCTGCCAATCAGT3′ and *usp*A R 5′ACGCAGACCGTAGGCCAGAT3′) was confirmed by the classical PCR method as reported by Chen and Griffiths (1998) [39]. The PCR master mix was prepared in 25 μL volume containing 5X reaction buffer (My Taq, Bioline, including MgSO_4_, dNTPs), 1 U of Taq DNA polymerase, 0.5 μL each of the forward and reverse primers, nuclease free water, and 5 μL of template DNA.

#### 3.3.2. ESBL-Encoding Gene Detection in *E. coli*

Isolates were evaluated for the presence of β-lactamase genes by multiplex PCR using the primers reported in Table 4.

For the PCR reaction mixture, 12.5 µL PCR Master mix, a 0.2 M concentration of each primer, and 2 µL template DNA were added to make the total volume 25 µL. The thermal-cycler temperature cycle was after an initial denaturation of 15 min at 95 °C, followed by 30 cycles of 30 s at 94 °C, 90 s at 62 °C, 60 s at 72 °C, followed by 72 °C for final extension. For agar gel electrophoresis performed to visualize the amplified products, 1X concentration Tris Acetic EDTA (TAE) and 1.5% agarose gel were used as buffer solution. For the isolates detected as positive for the *CTX-M* gene, a multiplex PCR reaction with a binding temperature of 55 °C was performed using the *CTX-M1*, *CTX-M2*, *CTX-M9*, and *CTX-M8/25* group primers, whose references are specified in Table 4.

#### 3.3.3. Sequence Analysis

The Sanger Gene sequence analysis method was performed for sequence analysis of the amplified products. With this sequencing method developed by Sanger et al. (1977), DNA sequences in the specific base range can be detected with high specificity by using fluorescent dye labelled dideoxy nucleotide triphosphates (ddNTP) [42]. The EurX GeneMATRIX Bacterial and Yeast DNA isolation kit (EURx, Gdansk, Poland) was used for DNA isolation for species level determination in the samples. Spectrophotometric measurement was performed on a Thermo Scientific Nanodrop 2000 (Thermo Fisher Scientific, Pittsburgh, PA, USA) device to control the amount and purity of the DNA samples. In the PCR study, targeted gene regions were amplified for species determination with 27F and 1492R primers as universal primers. Primer sequences 27F 5′ AGAGTTTGATCMTGGCTCAG 3′ and 1492R 5′ TACGGYTACCTTGTTACGACTT were used. PCR conditions were initial denaturation at 95 °C for 5 min followed by 45 s denaturation at 95 °C, 45 s annealing at 57 °C, 1 min extension at 72 °C (30 cycles), and final extension at 72 °C for 5 min. The amplification results obtained by PCR (Kyratec, SuperCycler Thermal Cycler, Wembley, Australia) were carried out using electrophoresis at 100 volts for 90 min on a 1.5% agarose gel prepared using 1X TAE buffer and ethidium bromide dye and were visualized under UV light. A one-step PCR process was applied to amplify the approximately 1470 base region.

The PCR reaction was carried out with the enzyme FIREPol^®^ DNA Polymerase Taq polymerase (Solis BioDyne, Tartu, Estonia). After PCR for the samples, a single band was obtained in the agarose gel and it was observed that the PCR process was successful. During the purification phase of the PCR product, the HighPrep™ PCR Clean-up System (MAGBIO AC-60005, MAGBIO, Kraichtal, Germany) purification kit was used for the single band samples obtained and each sample was purified in accordance with the manufacturer’s procedures. For the Sanger Sequencing samples, the ABI 3730XL Sanger sequencer (Applied Biosystems, Foster City, CA, USA) and the BigDye Terminator v3.1 Cycle Sequencing Kit (Applied Biosystems, Foster City, CA, USA) were used. The reads obtained with the primers 27F and 1492R were contig-formed to generate a consensus sequence. The CAP contig assembly algorithm was used in the BioEdit software (Version 7.2.5) to perform this process.

### 3.4. Phenotypic Antimicrobial Resistance Profile

The detection of antimicrobial resistance profiles in isolates was performed by Kirby-Bauer Agar Disk Diffusion method following the Clinical and Laboratory Standards Institute (CLSI) recommendations [43]. Ampicillin (AM; 25 µg), amoxicillin–clavulanic acid (AMC; 30 µg), aztreonam (ATM; 30 µg), cefepime (FEP; 30 µg), cefpodoxime (CPD; 30 µg), moxalactam (MOX; 30 µg), cefuroxime (CXM; 30 µg), cephalotine (KF; 30 µg), imipenem (IPM; 10 µg), and meropenem (MEM; 10 µg) antibiotic disks were used. The results were interpreted according to the European Committee on Antimicrobial Susceptibility Testing (EUCAST) guidelines and CLSI (2007) recommendations for moxalactam [43,44]. *E. coli* ATCC 25922 was used as the quality control strain. Phenotypic confirmation of ESBL production was carried out using the E-test and cefpodoxime combination disk method. Five samples, each representing the classical culture morphology of the identified isolates, tested positive for ESBL production. The reference strains of ESBL producing *E. coli* NCTC 14477 and ESBL producing *Klebsiella pneumoniae* NCTC 13440 were used as the positive control strains. Antimicrobial susceptibility testing was performed in triplicate for each isolate to ensure consistency and reliability of results.

### 3.5. Statistical Analysis

The relationship between seasons (cold and warm periods) and positivity rates was analyzed using Fisher’s Exact Test. This test evaluates the exact probability of observing the given distribution of positive and negative samples under the null hypothesis of independence. The analysis was conducted at a significance level of α = 0.05, and the resulting *p*-value was used to determine statistical significance. Statistical analyses were performed using SPSS version 21.00. Where appropriate, 95% confidence intervals were calculated to quantify uncertainty in the estimates. Given the sample size, a power analysis confirmed that the study was sufficiently powered to detect meaningful differences. Where applicable, odds ratios and 95% confidence intervals were calculated to assess the strength and precision of associations. Assumptions of the test were checked to ensure the robustness of results.

## 4. Conclusions

Our study is among the few investigating ESBL-producing *E. coli* in the laying hen slaughter and production environment. While most research has focused on broiler production, our findings highlight that the slaughter line, personnel, evisceration stage, and wastewater are key contamination points. The evisceration stage is particularly critical for the spread of antibiotic-resistant bacteria, and proper wastewater treatment should be a primary preventive measure.

Identifying contamination sources and mitigating their spread is essential, as restrictions on antibiotic use alone are insufficient to reduce global antimicrobial resistance. Therefore, implementing stricter regulations on antibiotic use in agriculture, particularly in poultry production, is critical to limiting the emergence and dissemination of resistant pathogens. Further longitudinal studies are needed to assess contamination dynamics and develop targeted control strategies within the laying hen production chain. In parallel, slaughterhouse operators should reinforce hygiene practices, especially during evisceration, and ensure proper wastewater management to limit bacterial spread. Moreover, addressing antimicrobial resistance in poultry production requires a One Health approach, recognizing the close relationship of animal, human, and environmental health. Collaborative efforts across sectors are crucial to minimizing bacterial transmission and protecting public health.

## Figures and Tables

**Table 1:** Sampling points of *ESBL* positive isolates.

Sample Points	Samples Taken for Analysis	January	February	March	June	July	August
Cloaca	C^1^	-	-	-	-	-	-
	C^2^	-	-	-	-	-	-
	C^3^	-	-	-	-	-	-
	C^4^	-	-	-	-	-	-
	C^5^	-	-	-	-	-	-
Personnel	P^1^	-	**+^1^**	-	-	-	-
	P^2^	-	-	-	**+^6^**	-	-
	P^3^	-	-	-	**+^7^**	-	-
	P^4^	-	-	-	-	-	-
	P^5^	-	-	-	-	-	**+^13^**
Air	slaughter line	-	-	-	-	-	-
	packaging–labeling	-	-	-	-	-	-
	cold storage warehouse	-	-	-	-	-	-
Wastewater	untreated	-	-	**+^3^**	-	**+^9^**	**+^14^**
	treated	-	-	-	-	-	-
Carcasses	after bloodletting^1^	-	-	-	-	**+^10^**	-
	after bloodletting^2^	-	-	-	-	-	-
	after evisceration^1^	-	-	-	-	**+^11^**	-
	after evisceration^2^	-	-	-	-	-	-
	after evisceration^3^	-	-	**+^4^**	-	-	**+^15^**
	after evisceration^4^	-	-	-	-	-	**+^16^**
Egg	egg^1^	-	**+^2^**	**+^5^**	-	-	**+^17^**
	egg^2^	-	-	-	**+^8^**	-	-
Final product	FP^1^	-	-	-	-	**+^12^**	-
	FP^2^	-	-	-	-	-	-

C: cloaca, P: personnel, and FP: final product. The numbers represent sample numbers; The numbers next to the bold text indicate the number of positives by month.

**Table 2:** Distribution of the ESBL genes across the sample groups.

Sample	*usp*A	bla*SHV*	bla*TEM*	bla*OXA*	bla*CTX-M*			
					bla*CTX-M1*	bla*CTX-M2*	bla*CTX-M9*	bla*CTX-M8/25*
Personnel 1	+	-	+	-	+	-	-	-
After evisceration 1	+	-	-	-	+	-	-	-
Raw wastewater 1	+	-	+	-	+	-	-	-
Personnel 2	+	-	-	-	+	-	-	-
Personnel 3	+	-	+	-	+	-	-	-
After evisceration 2	+	+	-	+	+	-	-	-
Raw wastewater 2	+	-	+	-	+	-	-	-
After evisceration 3	+	-	-	-	+	-	-	-
After evisceration 4	+	-	+	-	+	-	-	-
Raw wastewater 3	+	-	-	-	+	-	-	-

The numbers represent the number of samples.

**Table 3:** Resistance and susceptibility test results of the ESBL isolates.

Isolate	AMC	AM	ATM	FEP	CPD	CXM	KF	IPM	MEM	MOX
P^1^	**R**	**R**	**R**	**R**	**R**	**R**	**R**	S	S	**R**
P^2^	**R**	**R**	**R**	**R**	**R**	**R**	**R**	S	S	I
P^3^	**R**	**R**	**R**	**R**	**R**	**R**	**R**	S	S	I
P^4^	**R**	**R**	**R**	**R**	**R**	**R**	**R**	S	S	S
RW^1^	**R**	**R**	**R**	**R**	**R**	**R**	**R**	S	S	S
RW^2^	**R**	**R**	**R**	**R**	**R**	**R**	**R**	S	S	S
RW^3^	**R**	**R**	**R**	**R**	**R**	**R**	**R**	S	S	S
CAB^1^	**R**	**R**	**R**	**R**	**R**	**R**	**R**	S	S	S
CAE^1^	**R**	**R**	**R**	**R**	**R**	**R**	**R**	S	S	S
CAE^2^	**R**	**R**	**R**	**R**	**R**	**R**	**R**	S	S	**R**
CAE^3^	**R**	**R**	**R**	**R**	**R**	**R**	**R**	S	S	S
CAE^4^	**R**	**R**	**R**	**R**	**R**	**R**	**R**	S	S	I
FP^1^	**R**	**R**	**R**	**R**	**R**	**R**	**R**	S	S	S
E^1^	**R**	**R**	**R**	**R**	**R**	**R**	**R**	S	S	I
E^2^	**R**	**R**	**R**	**R**	**R**	**R**	**R**	S	S	S
E^3^	**R**	**R**	**R**	**R**	**R**	**R**	**R**	S	S	I
E^4^	**R**	**R**	**R**	**R**	**R**	**R**	**R**	S	S	I
**%R**	100	100	100	100	100	100	100	0	0	29.4
**%I**	0	0	0	0	0	0	0	0	0	35.3
**%S**	0	0	0	0	0	0	0	100	100	35.3

R: resistant, I: intermediate resistance, S: sensitive, AMC: amoxicillin–clavulanic acid, AM: ampicillin, ATM: aztreonam, FEP: cefepime, CPD: cefpodoxime, CXM: cefuroxime, KF: cephalothin, IPM: imipenem, MEM: meropenem, MOX: moxalactam, P: personnel, RW: raw wastewater, CAB: carcass after bloodletting, CAE: carcass after evisceration, FP: final product, and E: egg. The numbers refer to the number of samples.

**Table 4:** Primers used for the detection of the ESBL genes.

Target Gene	Primer Sequence (5′ to 3′)	Amplicon Size (bp)	Reference
bla*SHV*	CTTTATCGGCCCTCACTCAA AGGTGCTCATCATGGGAAAG	237	[40]
bla*TEM*	CGCCGCATACACTATTCTCAGAATGA ACGCTCACCGGCTCCAGATTTAT	445	[40]
bla*OXA*	ACACAATACATATCAACTTCGC AGTGTGTTTAGAATGGTGATC	813	[40]
bla*CTX-M*	ATGTGCAGYACCAGTAARGTKATGGC TGGGTRAARTARGTSACCAGAAYCAGCGG	593	[40]
bla*CTX-M1*	CGTCACGCTGTTGT TAGGAA TCGGTTCGCTTTCACTTTTC	227	[40]
bla*CTX-M2*	GGAGAAAAGTTCGGGAGGTC GCTTATCGCTCTCGCTCTGT	155	[40]
bla*CTX-M9*	ACGTGGCTCAAAGGCAATAC CGG CTG GGT AAA ATA GGT CA	174	[40]
bla*CTX-M8/25*	AACRCRCAGACGCTCTAC TCGAGCCGGAASGTGTYAT	326	[41]

## Data Availability

Restrictions apply to the availability of data used in this study. The obtained data are available from the authors with permission.

## References

[1] Wu Y., Wang C.H., Li X., Li F., Jiang M.L., Liu Z.K., Liu G.F., Li J.Y. (2024). Characteristics of the Plasmid-Mediate Colistin-Resistance Gene Mcr-1 in *Escherichia coli* Isolated from Pig Farm in Jiangxi. Pak. Vet. J..

[2] Xue M., Li Z., Zhang P., Lei W. (2024). Genomic Characteristics of ETT2 Gene Clusters in Avian Pathogenic *Escherichia coli* Identified by Whole-genome Sequencing. Pak. Vet. J..

[3] Kizil S., Aydin F.E., Önel A.U., Yildirim M., Güneri C.Ö., Cecen E.M. (2024). Determination of Subtypes, Serogroups, And Serotypes, Virulence, and/or Toxigenic Properties of *Escherichia coli* Isolated from Cattle, Sheep, and Goat Feces by Multiplex PCR. Kafkas Univ. Vet. Fak. Derg..

[4] Blaak H., van Hoek A.H.A.M., Hamidjaja R.A., van der Plaats R.Q.J., Kerkhof-de Heer L., de Roda Husman A.M., Schets F.M. (2015). Distribution, numbers, and diversity of ESBL producing *E. coli* in the poultry farm environment. PLoS ONE.

[5] Ungureanu V., Corcionivoschi N., Gundogdu O., Stef L., Pet I., Păcală N., Madden R.H. (2019). The emergence of beta-lactamase producing *Escherichia coli* and the problems in assessing their potential contribution to foodborne illness: A Review. AgroLife Sci. J..

[6] Lemlem M., Aklilu E., Mohammed M., Kamaruzzaman F., Zakaria Z., Harun A., Devan S.S. (2023). Molecular detection and antimicrobial resistance profiles of Extended-Spectrum Beta-Lactamase (ESBL) producing *Escherichia coli* in broiler chicken farms in Malaysia. PLoS ONE.

[7] Shaikh S., Fatima J., Shakil S., Rizvi S.M.D., Kamal M.A. (2015). Antibiotic resistance and extended spectrum beta-lactamases: Types, epidemiology and treatment. Saudi J. Biol. Sci..

[8] Falagas M.E., Karageorgopoulos D.E. (2009). Extended-spectrum ß-lactamase-producing organisms. J. Hosp. Infect..

[9] Urban-Chmiel R., Marek A., Stepien-Pysniak D., Wieczorek K., Dec M., Nowaczek A., Osek J. (2022). Antibiotic resistance in bacteria-a review. Antibiotics.

[10] Hu Y., Yang X., Li J., Lv N., Liu F., Wu J., Lin I.Y.C., Wu N., Weimer B.C., Gao G.F. (2016). The bacterial mobile resistome transfer network connecting the animal and human microbiomes. Appl. Environ. Microbiol..

[11] Xu C., Kong L., Gao H., Cheng X., Wang X. (2022). A review of current bacterial resistance to antibiotics in food animals. Front. Microbiol..

[12] Kwang-Won S., Young-Ju L. (2019). Detection of plasmid-mediated quinolone resistance genes in β-lactamase-producing *Escherichia coli* isolates from layer hens. Poult. Sci..

[13] Aldea I., Gibello A., Hernandez M., Leekitcharoenphon P., Bortolaia V., Moreno M.A. (2022). Clonal and plasmid-mediated flow of ESBL/AmpC genes in *Escherichia coli* in a commercial laying hen farm. Vet. Microbiol..

[14] Rahman A., Rahman Chowdhury M.S., Hossain H., Elsaid F.G., Almutairi L.A., Begum R., Sabrin M.S., Akanda A.R., Mukter Hossain M.M., Rafiqul Islam M.R. (2024). Identification of Virulence Genes and Multidrug Resistance in Shiga-Toxin Producing *Escherichia coli* (STEC) from Migratory and Captive Wild Birds. Pak. Vet. J..

[15] General Directorate of Meteorology (2023). Official Statistics: Provincial Seasonal Norms. https://www.mgm.gov.tr/veridegerlendirme/il-ve-ilceler-istatistik.aspx?m=KONYA.

[16] Khalid N., Bukhari S.M., Alshahrani M.Y., Rehman K.U., Ahmad S., Andleeb S., Javid A., Azam S.M. (2023). Nucleotide analysis and prevalence of *Escherichia coli* isolated from feces of some captive avian species. J. King Saud Univ. Sci..

[17] Wibisono F.J., Sumiatro B., Untari T., Effendi M.H., Permatasari D.A., Witaningrum A.M. (2021). Molecular identification of ctx gene of extended spectrum beta-lactamases (ESBL) producing *Escherichia coli* on layer chicken in Blitar, Indonesia. J. Anim. Plant Sci..

[18] Aliyu A.B., Jalila A., Saleha A.A., Zunita Z. (2024). ESBL Producing *E. coli* in Chickens and Poultry Farms Environment in Selangor, Malaysia: A Cross-Sectional Study on Their Occurrence and Associated Risk Factors with Environment and Public Health Importance. Zoonoses Public Health.

[19] Gazal L.E.D.S., Medeiros L.P., Dibo M., Nishio E.K., Koga V.L., Gonçalves B.C., Grassotti T.T., Leal de Camargo T.C., Pinheiro J.J., Vespero E.C. (2021). Detection of ESBL/AmpC-producing and fosfomycin-resistant *Escherichia coli* from different sources in poultry production in Southern Brazil. Front. Microbiol..

[20] Ewers C., De Jong A., Prenger-Berninghoff E., El Garch F., Leidner U., Tiwari S.K., Semmler T. (2021). Genomic diversity and virulence potential of ESBL-and AmpC-β-lactamase-producing *Escherichia coli* strains from healthy food animals across Europe. Front. Microbiol..

[21] Effendi M.H., Wibisono F.J., Witaningrum A.M., Permatasari D.A. (2021). Identification of blaTEM and blaSHV genes of extended spectrum beta lactamase (ESBL) producing *Escherichia coli* from broilers chicken in Blitar, Indonesia. Sys. Rev. Pharm..

[22] Faridah H.D., Wibisono F.M., Wibisono F.J., Nisa N., Fatimah F., Effendi M.H., Ugbo E.N., Khairullah A.R., Kurniawan S.C., Silaen O.S.M. (2023). Prevalence of the blaCTX-M and blaTEM genes among extended-spectrum beta lactamase-producing Escherichia coli isolated from broiler chickens in Indonesia. J. Vet. Res..

[23] Kawamura K., Nagano N., Suzuki M., Wachino J., Kimura K., Arakawa Y. (2017). ESBL-producing *Escherichia coli* and its rapid rise among healthy people. Food Saf..

[24] Overdevest I., Willemsen I., Rijnsburger M., Eustace A., Xu L., Hawkey P., Heck M., Savelkoul P., Vandenbroucke-Grauls C., van der Zwaluw K. (2011). Extended-spectrum β-lactamase genes of *Escherichia coli* in chicken meat and humans, the Netherlands. Emerg. Infect. Dis..

[25] Huijbers P.M.C., Graat E.A.M., Haenen A.P.J., van Santen M.G., Van Essen-Zandbergen A., Mevius D.J., van Duijkeren E., van Hoek A.H.A.M. (2014). Extended-spectrum and AmpCβ-lactamase-producing *Escherichia coli* in broilers and people living and/or working on broiler farms: Prevalence, risk factors and molecular characteristics. J. Antimicrob. Chemother..

[26] Harada S., Ishii Y., Yamaguchi K. (2008). Extended-spectrum β-lactamases: Implications for the clinical laboratory and therapy. Korean J. Lab. Med..

[27] Meng M., Li Y., Yao H. (2022). Plasmid-mediated transfer of antibiotic resistance genes in soil. Antibiotics.

[28] Wu Y., Huang S., Zhang D., Ji H., Ni Y., Zhang X., Dong J., Li B. (2023). Characteristics of Extended-Spectrum β-Lactamase-Producing *Escherichia coli* Derived from Food and Humans in Northern Xinjiang, China. Foodborne Pathog. Dis..

[29] Widodo A., Khairullah A.R., Effendi M.H., Moses I.B., Agustin A.L.D. (2024). Extended-spectrum β-lactamase-producing *Escherichia coli* from poultry: A review. Vet. World.

[30] Becker E., Projahn M., Burow E., Käsbohrer A. (2021). Are there effective intervention measures in broiler production against the ESBL/AmpC producer *Escherichia coli*?. Pathogens.

[31] Olopade A., Bitrus A.A., Momoh-Zekeri A.H., Bamayi P.H. (2022). Multi-drug resistant phenotypes of extended-spectrum β-lactamase (ESBL)-producing *E. coli* from layer chickens. Iraqi J. Vet. Sci..

[32] Pais S., Costa M., Barata A.R., Rodrigues L., Afonso I.M., Almeida G. (2023). Evaluation of antimicrobial resistance of different phylogroups of *Escherichia coli* isolates from feces of breeding and laying hens. Antibiotics.

[33] Wibisono F.J., Sumiatro B., Untari T., Effendi M.H., Permatasari D.A., Witaningrum A.M. (2020). Pattern of antibiotic resistance on extended-spectrum beta-lactamases genes producing *Escherichia coli* on laying hens in Blitar, Indonesia. Biodiversitas.

[34] Benameur Q., Gervasi T., Dahloum L., Rechidi-Sidhoum N., Benklaouz M.B., Yakubu A. (2023). Multidrug-resistant *Escherichia coli* isolated from cleaned and disinfected poultry houses prior to day-old chick placement. J. Environ. Qual..

[35] (2018). Microbiology of the Food Chain—Horizontal Methods for Surface Sampling.

[36] Hess-Kosa K. (2010). Indoor Air Quality: Sampling Methodologies.

[37] (2006). Water Quality—Sampling for Microbiological Analysis.

[38] Ongut G., Daloglu A.E., Baysan B.O., Daglar D., Ogunc D., Sekercioglu A.O., Colak D., Gunseren F. (2014). Evaluation of a chromogenic medium for detection of extended-spectrum-beta-lactamase-producing *Escherichia coli* and *Klebsiella pneumoniae* strains. Clin. Lab..

[39] Chen J., Griffiths M.W. (1998). PCR differentiation of *Escherichia coli* from other Gram-negative bacteria using primers derived from the nucleotide sequences flanking the gene encoding the universal stress protein. Lett. Appl. Microbiol..

[40] Fang H., Ataker F., Hedin G., Dornbusch K. (2008). Molecular epidemiology of extended-spectrum beta-lactamases among *Escherichia coli* isolates collected in a Swedish hospital an dits associated health care facilities from 2001 to 2006. J. Clin. Microbiol..

[41] Dallenne C., Da Costa A., Decre D., Favier C., Arlet G. (2010). Development of a set of multiplex PCR assays for the detection of genes encoding important b-lactamases in *Enterobacteriaceae*. J. Antimicrob. Chemother..

[42] Sanger F., Nicklen S., Coulson A.R. (1977). DNA sequencing with chain-terminating inhibitors. Proc. Natl. Acad. Sci. USA.

[43] CLSI Performance Standards for Antimicrobial Susceptibility Testing: Seventeenth Informational Suplement Approved Standard M100-S17. https://clsi.org/standards/products/microbiology/documents/m100/.

[44] EUCAST The European Committee on Antimicrobial Susceptibility Testing, Breakpoint Tables for Interpretation of MICs and Zone Diameters, Version 13.1. https://www.eucast.org/fileadmin/src/media/PDFs/EUCAST_files/Breakpoint_tables/v_13.1_Breakpoint_Tables.pdf.

